# Supplementary material for: The PRISMA 2020 Statement: A System Review of Hospital Preparedness for Bioterrorism Events
Source: Int J Environ Res Public Health. 2022 Dec 5;19(23):16257. doi: 10.3390/ijerph192316257 (PMC9737925; doi:10.3390/ijerph192316257)
Supplement: Supplementary file 1 [file ijerph-19-16257-s001.zip › ijerph-1986137-supplementary.pdf]

Supplementary File S1: China National Key Research and Development Program

No.	Objective	Design	Survey Participants/ Reference	Response Rate	Index of Composition	Discussion	Limitation
1	Investigate if there is a relationship among hospitals' preparedness for various emergency scenarios, and whether components of one emergency scenario correlate with preparedness for other emergency scenarios.	Cross-sectional	/	/	Statistical analyzed the relationship between overall hospital preparedness score for different emergency scenarios, between categories to total hospital preparedness score for the various emergency scenarios, SOPs, training and drills, knowledge of staff, and Infrastructure and equipment.	A well-developed SOP in mass toxicological events is vital. Training personnel and conducting drills are important factors of the emergency preparedness process. And training programs should include opportunities for staff to become adept in using equipment and infrastructure.	The evaluation was not include the preparedness of radiological events. This study does not answer to question of how often exercises and drills need to be held and address the degree of preparedness for one type of emergency actually enhances preparedness.
2	Establish an evaluation index system of military hospital's ability to deal with biological terrorism in accordance with national conditions and military situation	Documentary study and Delphi method	/	/	Five first-level indicators and 16 second-level indicators were determined including organization management, hospital treatment, pre-hospital first aid, support, education improvement.	Comprehensive bioterrorism capability requires the participation of multi-department managers	Due to the particularity of military hospitals, the survey results are not universal

3	Improve the capability military hospitals for bioterrorism response.	Cross-section al	134	82.09%	The questionnaire is divided into 6 parts, including basic situation of the hospital, organizational management ability to deal with bioterrorism, pre-hospital emergency ability, in-hospital treatment ability, support ability and education improvement ability.	Equipment, biological protection, diagnosis and isolation treatment, biological pollution of the environment detection and disinfection specific disposal ability remains to be improved.	The survey did not classify the selected hospitals according to their characteristics.
4	Hospital disaster preparedness in Switzerland	Cross-section al	138	88%	Investigate the disaster planning, surge beds and human resource.	The preparation of an international event may provide an incentive for State Health services to control the existence and functionality of hospital disaster plans. Facility-based surge capacity, drill	The study describes the information in 2006 may be outdated. And covered only 88%, it cannot assess whether their characteristics differed significantly.

5	Investigates the hospital disaster preparedness in Jeddah.	Cross-sectional	8	75%	Questionnaire was divided into eight fields of 33 indicators: structure, architectural and furnishings, lifeline facilities safety, hospital location, utilities maintenance, surge capacity, emergency and disaster plan, control of communication and coordination.	Hospital lack training and management. Weaknesses were apparent particularly in the following indicators: Emergency and Disaster plan; especially in Hazard map, Information, Committee, Preparedness, Emergency Incident Command System, and Stage of activation, Emergency operations center and Surveillance system. Hospitals also have showed weakness in control of communication and coordination and its sub indicators	Results were based on only one respondent from each hospital. Therefore, the respondent's feedback may not reflect accurately the hospital policy. The restriction of the data collection to a single city limits the generalization of the results.
6	Establish a comprehensive evaluation system for military hospitals' response capacity to bioterrorism	Documentary study, Cross-sectional and Delphi method	134	100%	Establish a Comprehensive Evaluation Index System for military hospitals' response capacity to bio-terrorism., including medical response, emergency management, consciousness, hardware support and improvement.	Factor analysis is a comprehensive evaluation method. The hospitals' response capability to bioterrorism is a comprehensive capability, military hospitals are essential in providing medical and psychological services.	The evaluation indicator system was not used to analyze the current situation of the response of military hospitals to bioterrorism.

7	Hospital Disaster Preparedness in Italy: a preliminary study utilizing the World Health Organization Hospital Emergency Response Evaluation Toolkit.	Cross-sectional	30	50%	WHO hospital emergency response checklist was used as the evaluation toolkit.<br>it is composed of 92 priority action items grouped in 9 key components, such as triage, surge capacity and critical services.	Hospital need to follow an implement standardized strategy.<br>The allocation of financial resources, communication, hospital resiliency and structural and non-structural safety, human resources and logistics are important.	The Sample size is too small. Another is that no evaluator participated in all of the assessment visits, inter-rater reliability was not measured.
8	Evaluate hospitals' disaster plans in the city of Makkah.	Cross-sectional	17	82%	The study surveyed the characteristics of all hospitals, surge capacities of the hospitals, staff knowledge and training and general evaluation of disaster plan.	The triage and management protocol, surgy capacity, staff benefit, education, investment and cooperative drills between hospitals would enhance resiliency of the region.<br>The plans should be as comprehensive as possible to cover all hazards and susceptible populations including pediatric, obstetric and geriatric populations.	Cover a specific area with a single sample resulted in a smaller sample size than anticipated. The data was difficult to assessing the quality and efficacy of the disaster plans described.

9	Assess the preparedness of Dutch hospitals for a large-scale CBRN event	Cross-section al	93	67%	Questions comprising hospital disaster planning, decontamination and PPE, antidotes, nuclear threats, biological threats. The size of a hospital and its university status did not significantly influence preparedness.	Financial aspects of paying for adequate preparedness in growing financial pressures on hospital's resources represent a major obstacle. Preparedness is a huge financial burden, which may cut costs when funds are limited.	This study is based on self-reported data and have a degree of reporting bias. And only a third of the hospitals responded, results may be higher than the actual level.
10	Survey of Biological Incidents Preparedness of Hospitals in Markazi Province in 2016	Cross-section al	19	100%	The data collection tool was the American Hospital Association Chemical and Bioterrorism Preparedness Checklist that contained 120 questions..	Organizing, support and logistics, process of medical treatments, education, hospital management and security, psychiatric services, and diagnosis are essential for bioterrorism preparedness.	The sample size is too small to be representative and checklists are difficult to localize.

11	Assess the disaster preparedness of hospitals in Qazvin.	Cross-section al	6	100%	The survey was assessed in functional capacity (plan, organization, medicines, supplies, instruments, and other equipment), structural (previous events affecting, safety of structural system), and nonstructural safety (electric, water, telecommunications, fuel storage, medical gases, furnishings, architectural elements).	It should develop non-structural components and a systematic short-term training programs in the field of crisis management for hospital administrator, other officials and experts.	The experts in each filed were required to complete high number of questions in the checklist.
12	A Study of Hospital Disaster Preparedness in South Yemen	Cross-section al	10	100%	Survey in nine components: (1) command and control; (2) communication; (3) safety and security; (4) triage; (5) surge capacity; (6) continuity of essential services; (7) human resources; (8) logistics and management supply; and (9) post-disaster recovery.	Human resources, triage, command and control, communication system with sustainable back-up resources, early warning, surveillance, and ventilation systems, training, mental health support and funds played a contributory role.	This study is the limited sample size. Another limitation is the validity of the surveying tool since there is no standardized instrument for assessment of hospital preparedness;

13	Systematically review the current research knowledge on hospital preparedness tools used in biological events and factors affecting hospital preparedness.	Systematic reviews and Meta-Analyses	/	/	Factors that were identified in the study to hospitals preparedness in biological events classified in seven areas including planning, surge capacity, communication, training and education, medical management, surveillance and standard operation process.	The available tools lack appropriate psychometrics, and none of the available tools has evaluated all the dimensions regarding the preparedness of hospitals in such events.	The study did not take into account actual hospital conditions.
14	Propose a hybrid fuzzy decision making model to evaluate the disaster preparedness of hospitals.	Documentary study and Delphi method	/	/	Apply a model FAHP to determine weights of six main criteria of hospital buildings, equipment, communication, transportation, personnel, flexibility and a total of thirty-six sub-criteria. TOPSIS is used to obtain ranking of hospitals.	The main weaknesses detected in the cited set of hospitals: 1) low availability of medical equipment, 2) lack of helipad spaces, 3) low availability of contingency staff, 4) lack of tents, and 5) low number of disaster management training programs.	The limitations are from methodological and application viewpoints Application viewpoint is limited with four Turkish hospitals.
15	Factors affecting hospital response in biological disasters: A qualitative study	Delphi method	12	100%	Inteview 12 emergency management experts and extract eight accepts including detection; treatment and infection control; coordination, resources; training and exercises; communication and information system; construction; and planning and assessment. .	Biologic triage, risk management approaches for infectious disasters, decontamination measures and surveillance, funding and financial resources are necessary.	The validity of the extracted indicators has not been verified

16	Hospital Preparedness Challenges in Biological Disasters: A Qualitative Study	Cross-sectional	20	100%	Inteview 20 emergency management experts and extract six accepts including education and training, resource management, patient management, risk communication, safety and health, laboratory, and surveillance.	Preparedness plan, providing various strategies including effective training; management of resources; safety system deployment; risk perception for managers, staff, timely informing; syndromic surveillance system; laboratory detection capacity and patient management can improve preparedness.	The generalization of the results of this study is limited only to the study environment
17	Assessing the preparedness of hospitals facing disasters using the rough set theory: guidelines for more preparedness to cope with the COVID-19	Cross-sectional	25	100%	The survey was assessed in crisis management framework, planning, insurance coverage, event management system, public services, education, and manure.	Adequate medical equipment, emergency medicine, sufficient bed space, and enhanced medical information system are required to be at a high level.	The study is based on the COVID-19 pandemic and cannot fully represent biological events

18	Hospital Disater Preparedness In Iranian Province: A Cross-Sectional Study Using A Standard Tool	Cross-section al	15	83%	WHO Hospital Emergency Response Checklist was used as an evaluation tool. It consists of nine key components: command and control, triage, human resources, communications, surge capacity, logistics/supply management, safety and security, continuity of essential services, and post-disaster recovery.	The logistics and essential services need to be focused.	The sample results are not represe-ntative of Iran as a whole.
19	Evaluate the current disaster preparedness status of the EMS agencies in the literature and exploring the key preparedness elements and the strategies to improve the EMS disaster preparedness.	Documentary study	/	/	The most important elements of the EMS disaster preparedness include the size and scope of the incident, surge capacity, planning, communication, training and education, policymaking, financial support, coordination, safety and security, early warning system, disaster response experience, and legal considerations.	The assessment tools used in the current study were not standard. Most of tools did not have psychometric analysis to confirm their validity. This highlights the necessity of developing standard all-hazard approach tools that have been validated through the appropriate psychometric process and measure comprehensively all aspects and dimensions of EMS agencies' preparedness in disasters.	Only the studies published in the English language were selected. And the inability to generalize the study results to all EMS agencies in the world due to lack of broad representation of most world nations about EMS disaster preparedness.

20	COSMIN Checklist for Systematic Reviews of the Hospital Preparedness Instruments in Biological Events	Systematic review and Meta-Analyse s	20	/	Disaster planning for hospitals, emergency command systems, nursing capacity, disinfectant showers, antibiotic stockpiles, cross-departmental and institutional mutual aid agreements	Hospital's preparedness was varied with hospital level, size and location. It's essential to develop an instrument with acceptable psychometric properties for measuring hospital preparedness in biological events.	The literature analysis lacks practical verification.
21	Investigating the level of functional preparedness of selected Tehran hospitals in the face of biological events: a focus on COVID-19	Cross-section al	4	100%	Five aspects of the hospital were surveyed: surge capacity, communication, biological consultants, meeting management and post-disaster recovery.	Recurrence of disease needs to be considered to increase hospital surge levels.	The Sample size is too small and was taken only in Tehran. The results are not universal.
22	Chemical, Biological, Radiological, or Nuclear Response in Queensland Emergency Serv ices: A Multisite Study	Cross-section al	7	100%	Investigate the hospitals' incident command or management system, surge capacity, staff support policy, training and exercise, electronic medical records and patient management.	Hospitals and clinical leaders must anticipate and be prepared for a surgeresponse. Decontamination support, education, training, and policies. The pediatric emergency care response should be at the forefront.	The sample is geographically restricted, which reduces the transferability of the findings. Some selected hospitals were unable to complete the survey items.

23	Establishing the Domains of a Hospital Disaster Preparedness Evaluation Tool: A Systematic Review	Systematic review	53	/	The study reviewed literatures across the world using the 4S framework- space, stuff, staff, and systems.	A safe site and accessibility are important aspects of a hospital disaster preparedness.Transport facilities, electric power, hospital stockpile, morgue facilities and dead body handling, vaccination, rewards/incentive,and volunteer themes, waste management should be considered.	This study selected only the published articles on hospital-based disaster preparedness studies and articles written in the English language from 2011-2020.
